# A Systematic Literature Review of the Use of Computational Text Analysis Methods in Intimate Partner Violence Research

**DOI:** 10.1007/s10896-023-00517-7

**Published:** 2023-03-21

**Authors:** Lilly Neubauer, Isabel Straw, Enrico Mariconti, Leonie Maria Tanczer

**Affiliations:** University College London, Gower Street, London, WC1E 6BT UK

**Keywords:** Intimate Partner Violence, Domestic Violence, Text Mining, Text Analysis, Machine Learning, Natural Language Processing, Systematic Review

## Abstract

**Purpose:**

Computational text mining methods are proposed as a useful methodological innovation in Intimate Partner Violence (IPV) research. Text mining can offer researchers access to existing or new datasets, sourced from social media or from IPV-related organisations, that would be too large to analyse manually. This article aims to give an overview of current work applying text mining methodologies in the study of IPV, as a starting point for researchers wanting to use such methods in their own work.

**Methods:**

This article reports the results of a systematic review of academic research using computational text mining to research IPV. A review protocol was developed according to PRISMA guidelines, and a literature search of 8 databases was conducted, identifying 22 unique studies that were included in the review.

**Results:**

The included studies cover a wide range of methodologies and outcomes. Supervised and unsupervised approaches are represented, including rule-based classification (*n* = 3), traditional Machine Learning (*n* = 8), Deep Learning (*n* = 6) and topic modelling (*n* = 4) methods. Datasets are mostly sourced from social media (*n* = 15), with other data being sourced from police forces (*n* = 3), health or social care providers (*n* = 3), or litigation texts (*n* = 1). Evaluation methods mostly used a held-out, labelled test set, or k-fold Cross Validation, with Accuracy and F1 metrics reported. Only a few studies commented on the ethics of computational IPV research.

**Conclusions:**

Text mining methodologies offer promising data collection and analysis techniques for IPV research. Future work in this space must consider ethical implications of computational approaches.

Existing methods for studying intimate partner violence (IPV) draw largely from the social sciences. These include primary data collection tools such as surveys (Lagdon et al., 2022; ONS, 2020), interviews or focus groups (Øverlien et al., 2020; Wood et al., 2021), as well as secondary analyses of data sourced from, for example, victim advocacy organisations (Rogers et al., 2019).

Recent developments in the field of *computational social science* have led to data science tools which extend and complement these established techniques (DiMaggio, 2015; Evans & Aceves, 2016). They further ease the data collection and analysis process by harnessing big data and Machine Learning (ML) (Gauthier & Wallace, 2022). The latter is a subset of artificial intelligence focused on building algorithms that ‘learn’ statistical patterns from large amounts of data.

Specifically, computational *text analysis* or *text mining* – umbrella terms for computational tools which can extract and analyse substantial quantities of text data – have been successfully utilised in fields such as social work (Victor et al., 2021), medicine (Luque et al., 2019), and education (Ferreira‐Mello et al., 2019). Indeed, a small number of studies have applied similar approaches to the study of IPV. Publications examined online support-seeking behaviours of victim-survivors (Chu et al., 2021), studied reasons given for staying and leaving abusive relationships in microblog posts (Homan et al., 2020), and identified crisis posts on social media platforms such as Facebook (S. Subramani et al., 2018a, 2018b). In addition, computational methods have offered IPV researchers access to datasets which are simply too large to evaluate manually e.g. police incident reports (J. Poelmans et al., 2009a, 2009b, 2009c, 2009d; Wilson et al., 2021), case summaries (Victor et al., 2021), and Electronic Health Records (Botelle et al., 2022).

Despite this small but growing body of work, there is yet no review addressing the application of computational text analysis methods to the study of IPV. This omission stands in the way of proposing further methodological innovation, and to opening the field to the latest transdisciplinary research approaches stemming from computer science. This article seeks to fill this gap by conducting a systematic literature review of eight online academic databases (Scopus, ProQuest, Web of Science, IEEE Explore, PsychInfo, PubMed, ArXiv.org and ACM Digital Library).

The rest of the article is structured as follows: 1) Background: a short background to both IPV and text mining is provided to give context to later discussions. 2) Research Questions (RQs): Several RQs are proposed to investigate the use of text mining methods in the IPV domain. 3) Methodology: The methodology of this review is described, including the search strategy and inclusion criteria. 4) Results: The results of the review are summarised and analysed using a 21-item checklist. 5) Discussion: The findings from the review, its limitations, and potential directions for future work are discussed. 6) Concluding remarks.

## Background

Data from the World Health Organisation (2021) indicates that 27% of women worldwide aged 15–49 years who have been in a relationship have experienced some form of physical or sexual violence from an intimate partner during their lifetime. The Crime Survey for England and Wales in 2020 indicated that 4.9% of women and 2.1% of men over the age of 16 had experienced some form of non-sexual partner abuse in the last year (ONS, 2020).

Despite these figures, accurately quantifying the prevalence of IPV is difficult (Walby et al., 2017). Much abuse goes unreported due to shame, bias, and unawareness (Stark, 2009). An additional barrier to measuring IPV is a non-homogenous set of definitions for what constitutes abuse across cultures, time periods, and organisations (Alhabib et al., 2010; Barocas et al., 2016). Whilst IPV is generally understood to involve physical abuse, there are other ways in which perpetrators cause harm (psychological, sexual, coercive controlling, economic, technology-facilitated abuse etc.). These alternative abuse forms may or may not be included in definitions, resulting in skewed evaluations (Alhabib et al., 2010; Dokkedahl et al., 2019).

Much of the existing large-scale data about IPV is drawn from traditional survey- and questionnaire-based research (Australian Bureau of Statistics, 2013; European Union Agency for Fundamental Rights, 2014). Whilst such surveys are useful to understand IPV on a population level, they are also costly, infrequent, and unlikely to capture granular data (Australian Bureau of Statistics, 2013). In this context, researchers often turn to interview-based approaches (Houston-Kolnik & Vasquez, 2022; Vatnar & Bjørkly, 2008). Although valuable, one-on-one interviews may also suffer from selection-bias, sample size issues, and being time-consuming to run (Karystianis et al., 2022).

Against this backdrop, some IPV researchers are turning to secondary analysis of existing data (Australian Bureau of Statistics, 2013). Organisations that interact with victim-survivors – such as police forces or health services – collect large quantities of IPV data which they are unable to analyse manually (Botelle et al., 2022; Karystianis et al., 2022). Additionally, victim-survivors of IPV increasingly make use of online venues such as blogs and bulletin boards to express their experiences of abuse and to receive and offer support (Chu et al., 2021; S. Subramani et al., 2019). These entries generate huge amounts of text data, much of which is publicly accessible.

Computational text mining is a set of techniques which use algorithms to understand, categorise or extract information from unstructured text data (DiMaggio, 2015). These can range from *simple* (for example, counting the occurrences of a pair of words in a corpus (Homan et al., 2020)) to *complex* approaches (for example, Deep Learning classifiers which use many layered neural networks to automatically categorise texts (S. Subramani et al., 2019)). Computational text mining methodologies have been used to harness big data to research social phenomena in other domains, such as the study of online hate (Fortuna & Nunes, 2018), cyberbullying (Rosa et al., 2019) and child abuse victimisation (Shahi et al., 2021). Given the intersection between these domains, plus the existing methodological issues in IPV research, computational text mining methodologies offer a promising avenue for the study of IPV.

## Research Questions

This article offers a systematic review of existing work which has applied computational text mining to the study of IPV. In doing so, it aims to provide a resource for IPV scholars who may want to use computational text methodologies in their work, providing a starting point to understand current capabilities as well as directions for future research. The article gives an introductory background to text mining methods and techniques, whilst seeking to examine the quality of current work. The authors do not assume existing knowledge of computational methodology, and all terminology will be explained within our article.

Our assessment of the academic literature is driven by three research questions: (RQ1) How have computational text analysis methods been *used* in IPV research?; (RQ2) What datasets are *available* for studying IPV using computational text analysis?; (RQ3) How have text analysis methods been *evaluated* in the study of IPV?

## Method

A systematic review of existing academic literature was conducted according to PRISMA-P guidelines (Moher et al., 2015).

### Electronic Search Strategy

Eight databases (ACM Digital Library, ArXiv.org, IEEE Xplore, ProQuest, PsychInfo, PubMed, Web of Science, Scopus) were searched, in March 2022, for all records containing *both* terms relating to computational text mining *and* terms relating to intimate partner violence, within all fields apart from the full-text (e.g. Title, abstract, keywords, publication venue), and unrestricted by date. The full search string was as follows^1^:

*((“artificial intelligence” OR “machine learning” OR “supervised learning” OR “unsupervised learning” OR “automatic detection” OR “automatic recognition” OR “text mining” OR “natural language processing” OR “deep learning” OR “text analysis” OR “information retrieval” OR “information extraction” OR “machine reading” OR “word embeddings” OR “feature extraction” OR “knowledge discovery” OR “data engineering” OR “knowledge engineering” OR “exploratory data analysis” OR “quantitative content analysis” OR “automatic content analysis” OR “computational methods” OR “big data” OR “predictive model”) AND (“intimate partner violence” OR “intimate partner abuse” OR “domestic violence” OR “domestic abuse” OR “family violence” OR “family abuse”))*

### Inclusion Criteria

Studies were included in the review if they met the following criteria:

- Peer reviewed and pre-print academic literature;
- The study uses computational text analysis or text mining to address an IPV-related outcome from a dataset which includes unstructured text fields;
- The study includes results from at least one dataset (studies which discuss a purely theoretical design or prototype were excluded);
- The main outcome of the computational model is related to the identification of types, characteristics, prevalence, behaviours and/or opinions of IPV (We excluded studies where IPV is used as an input feature rather than an outcome, for example studies measuring the impact of IPV (input) on mental health (outcome));
- Since IPV is defined differently in different research, and sometimes is captured within other definitions of violence, we included studies with “family violence” “domestic violence” or “sexual violence” related outcomes, since these may include IPV within their definitions.

### Data Extraction and Management

Records identified through database searches were imported into Rayyan (Ouzzani et al., 2016) for data management. After duplicates had been discarded, two of the authors independently performed abstract screening according to the above inclusion criteria. Cohen’s Kappa statistic was calculated at this stage to determine Inter Rater Reliability (IRR) following the procedure described by Hallgren (2012). Cohen’s Kappa was 0.69, indicating a substantial level of agreement between the two reviewers, according to guidelines from Landis and Koch (1977). Remaining disagreements were resolved following a discussion between the two reviewers.

The included papers were subsequently downloaded and a pro-forma was used to extract the information from each paper. The pro-forma was piloted with 16 initial papers and feedback was obtained from other authors, following which amendments were made. The final pro-forma consisted of the following information fields:

*Authors; Name of study; Year of study; IPV-related hypothesis or outcome; Source, size and time period of dataset; Demographics of dataset (if discussed); Method and results of labelling dataset; Data pre-processing and cleaning process (if mentioned); Feature selection process (if mentioned); Model task; Types of models tested; Best performing model; Evaluation method; Evaluation metrics used; Best evaluation outcome; Summary of discussion of evaluation outcomes (if any); Summary of interpretability of the model (if discussed); Technologies mentioned; The definition of violence used by the study (if any); Summary of ethical discussion or limitations (if any); Whether any code/datasets are open source.*

### Quality Assessment

Existing guidelines for assessing bias, quality, and reliability of biomedical or psychological studies are difficult to apply to research using computational text-analysis methods, particularly when reviewing highly specialised systems such as those involving ML. This paper builds on existing frameworks for assessing ML and mixed methods research (Dreisbach et al., 2019; Hinds et al., 2021; Hong et al., 2018; Siebert et al., 2020) to develop a checklist of 21 ‘yes/no’ criteria which were used to assess the overall quality, reliability and potential bias of studies included in the review. A wide range of approaches are surveyed in the included studies, so some irrelevant items were excluded from the checklist depending on the study in question. For that reason, the checklist is not supposed to provide a ranking of studies but an indication of overall quality of the included works. The 21-item checklist was as follows:

1. Definition of violence discussed
2. Clearly described and motivated IPV-related hypothesis or outcome
3. Representativeness/demographics of dataset discussed and/or analysed
4. Source, size, and time period of dataset reported
5. Data cleaning and sampling process reported
6. Discussion of pre-processing techniques
7. Appropriate model used for hypothesis
8. Feature selection discussed and/or different features considered
9. Different models tested and compared
10. Clear and appropriate evaluation criteria
11. Evaluation outcomes reported
12. Evaluation outcomes discussed e.g. comparison to other work, discuss misclassifications
13. Study includes discussion of model interpretability, or clearly explains model rules
14. Includes ethical discussion
15. Source code and/or datasets available
16. Includes discussion of limitations of model and/or appropriate use
17. Dataset is of an appropriate size, and balance of classes discussed
18. Data labelling process is explained
19. Data is labelled according to a protocol by more than one annotator and IAA reported
20. Model is tested on held-out ‘test’ set
21. Model is tested or deployed “in the wild”

## Results

### Included Studies

**Fig. 1:**
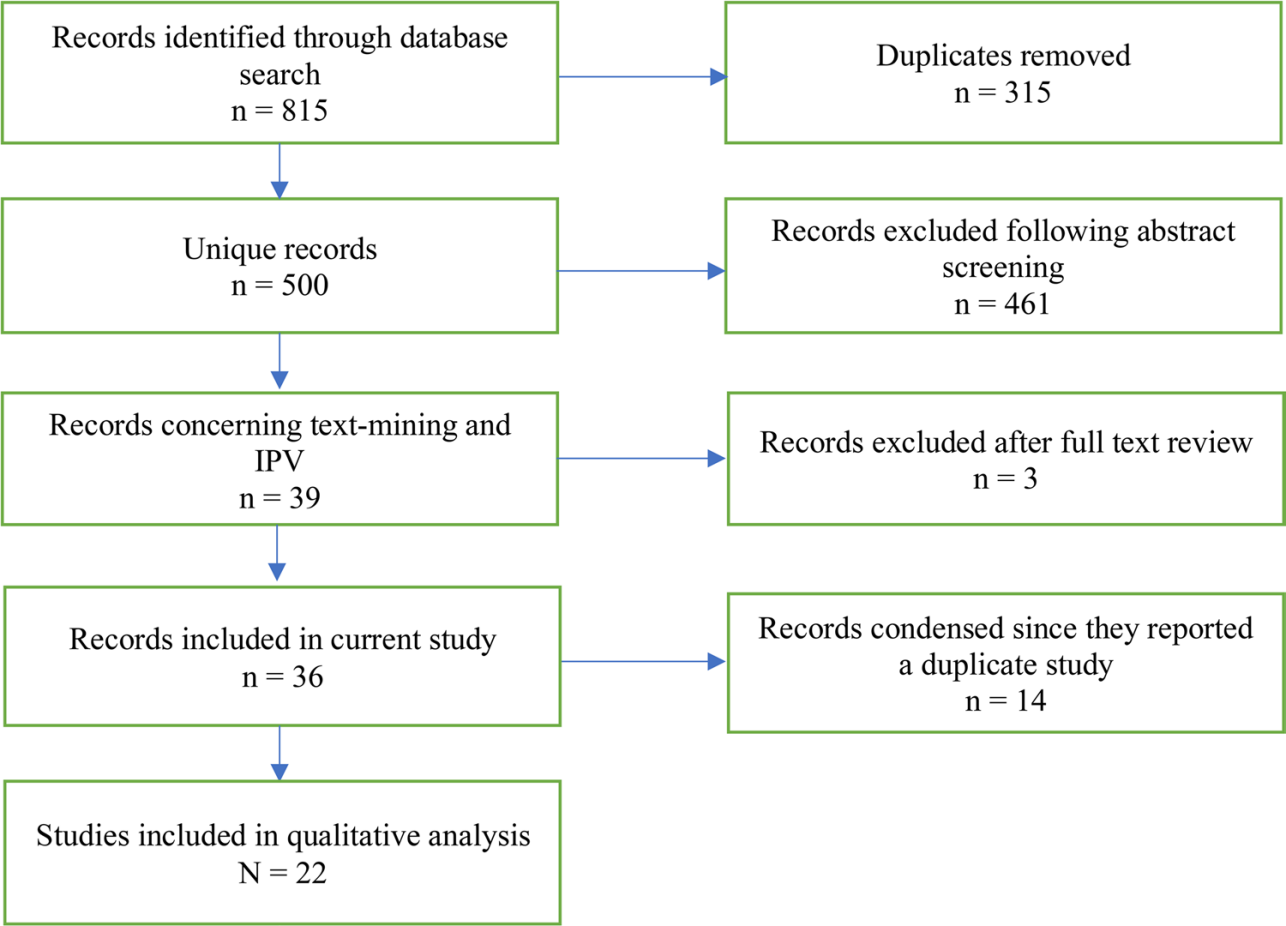
PRISMA Chart

As can be seen in the PRISMA chart in Fig. 1, the search yielded 815 results of which 315 were duplicates, leaving 500 unique studies. Of these, 461 were excluded as irrelevant (meaning they did not mention intimate partner abuse and/or use a computational text mining methodology) during abstract screening, leaving 39 papers.

Following full text review, a further three records were excluded because: no full text was available (*n* = 1); the text was not written in English (*n* = 1); the paper discussed a purely theoretical approach which did not involve any data (*n* = 1). Finally, a number of papers (*n* = 16) were found to report on the same two broad studies, using similar datasets and models. These were the Karystianis et al. papers on the New South Wales Police Force data using a rule-based approach, *n* = 6 (Adily et al., 2021; Hwang et al., 2020; Karystianis et al., 2019, 2022; Wilson et al., 2021; Withall et al., 2022), and the Poelmans et al. papers on the Amsterdam-Amstelland Police Force Data using an FCA and ESOM based approach, *n* = 10 (Elzinga, Poelmans, Viaene, & Dedene, 2009; J. Poelmans, Elzinga, & Dedene, 2013; J. Poelmans, Elzinga, Viaene, & Dedene, 2008, 2009; Jonas Poelmans et al., 2010; J Poelmans et al., 2011a, 2011b; J. Poelmans, Elzinga, Viaene, Dedene, & Van Hulle, 2009; J. Poelmans, Elzinga, Viaene, Hulle, et al., 2009a, 2009b, 2009c, 2009d; J. Poelmans, Elzinga, Viaene, Van Hulle, & Dedene, 2009a, 2009b, 2009c, 2009d; J Poelmans et al., 2011a, 2011b)). For simplicity of reporting in this review, these records were condensed into two unique studies. This left *N* = 22 unique studies to be included in the following qualitative analysis. A summary of the included studies can be found in Table 1.

**Table 1:** Summary of Included Studies

Name of Study	Authors	Year	Dataset	Model Task	(Most Successful) Model Type	Evaluation Outcome (of best performing model)
Public Attention and Sentiment toward Intimate Partner Violence Based on Weibo in China: A Text Mining Approach	Xu et al.	2022	Chinese Social Media Comments about Yuya IPV disclosure	Unsupervised Sentiment Analysis	Custom rule-based sentiment analysis algorithm using a combination of pre-trained and custom-built dictionaries	N/A—unsupervised classification
Can Natural Language Processing Models Extract and Classify Instances of Interpersonal Violence in Mental Healthcare Electronic Records: an Applied Evaluative Study	Botelle et al.	2022	Electronic Health Records (EHRs) from the South London and Maudsley NHS Foundation Trust	Seven binary classification tasks: presence/absence of violence, patient status (victim, perpetrator and/or witness) and violence type (domestic, physical, sexual)	Pre-trained BioBERT Model	Tenfold Cross-Validation (CV); F1 score > 0.89. Best F1 = 0.97 for sexual violence
Analyzing the Impact of Domestic Violence on Social Media using Natural Language Processing	More and Francis	2021	English-Language Reddit posts, Tweets and news articles discussing domestic abuse	Unsupervised Topic Modelling	Latent Dirichlet Allocation (LDA) and other unsupervised topic models	Topic coherence—results not discussed
Utilizing Text Mining, Data Linkage and Deep Learning in Police and Health Records to Predict Future Offenses in Family and Domestic Violence	Karystianis et al.	2021	Police-attended domestic violence event narratives from New South Wales, Australia. This data was linked with data about mental health diagnosis, age at diagnosis, episode start and end data, from the New South Wales Ministry of Health	Multi-class time series prediction model (Predict probability of future offense in three categories: physical, non-physical, Apprehended Domestic Violence Order (ADVO) breach)	Transformer model with BERT embeddings	Best accuracy = 0.69 for predicting *ADVO breach* in multi-class classification
A Computational Social Science Perspective on Qualitative Data Exploration: Using Topic Models for the Descriptive Analysis of Social Media Data	Rodriguez and Storer	2020	English-Language Tweets tagged with #WhyIstayed or #WhyILeft	Unsupervised Topic Modelling	Structural Topic Model with 65 topics	Held-out Likelihood, Residuals, Semantic Coherence, and Maximised Lower Bound used to evaluate goodness of fit for clustering—65 topics identified as best fit according to these metrics
Using Data Mining Techniques to Examine Domestic Violence Topics on Twitter	Xue et al.	2019	Tweets about Domestic Violence	Unsupervised Topic Modelling	LDA with 20 topics	Rate of Perplexity Change (RPC) used to evaluate best number of topics
Sexual Harassment Story Classification and Key Information Identification	Liu et al.	2019	Safecity English-language narratives of sexual harassment incidents (originally published by Karlekar and Bansal)	Entity Recognition (four entity types: harasser, time, location, trigger words); Multi-class classification (5 story types)	Convolutional Neural Network (CNN)	Accuracy = 0.92 for Entity Recognition. Highest accuracy for multi-class classification was 0.97 achieved by a CNN model for "Time of Day"; for "type of harasser" this was 0.93
Apply Event Extraction Techniques to the Judicial Field	Li et al.	2019	Chinese-Language litigation texts from divorce proceedings	Event Extraction (13 types of event in divorce cases, one of which is an event of domestic violence)	Combined architecture consisting of dictionary methods and 2 × Long ShortTerm Memory (LSTM) and Conditional Random Field (CRF) models	Tenfold CV; F1 = 0.84
Understanding the Silence of Sexual Harassment Victims through the #WhyIDidntReport Movement	Garrett and Hassan	2019	Tweets using the #WhyIDidntReport hashtag from US cities	Multi-class classification (8 different reasons for not reporting sexual violence e.g. shame, feeling hopeless, lack of information, protecting perpetrator etc.)	Support Vector Machine (SVM)	F1 ranged from 0.47—0.78 across different classes
Corpus-driven Insights into the Discourse of Women Survivors of Intimate Partner Violence	Sanchez-Moya	2017	British domestic violence charity online forum posts	Linguistic Analysis	Linguistic Inquiry and Word Count. (LIWC)	N/A
An Analysis of Domestic Abuse Discourse on Reddit	Schrading et al.	2015	Reddit posts discussing abuse and not relevant to abuse	Binary Classification (About abuse / not about abuse)	Linear SVM	Accuracy = 0.92 was the best performance achieved on a train/test set of submissions concatenated with their topscoring comments. Also tested 'in the wild' on Reddit data from general 'relationship advice' forums
Indirect Identification of Perinatal Psychosocial Risks from Natural Language	Allen et al.	2021	Diary entries of pregnant women	Binary Classification (IPV—non-IPV)	LASSO Regression with sentiment, topic modelling and LIWC as features	Regression R^2, Area Under Curve = 0.08, 0.75 (test set)
Online Social Support for Intimate Partner Violence Victims in China: Quantitative and Automatic Content Analysis	Chu et al.	2021	Chinese-Language posts from Baidu Teiba group about intimate partner violence	Multi-class Classification (Emotional Support, Informational Support or None)	Logistic Regression	Classification Accuracy = 0.94; F1 = 0.56 (test set)
Deep Learning for Multi-Class Identification From Domestic Violence Online Posts	Subramani et al.	2019	Facebook posts relating to domestic violence	Multi-class Classification (Type of Support)	CNN and LSTM-type models; SVM with Term Frequency-Inverse Document Frequency (TF-IDF) vectors as features	Classification Accuracy = 0.91; F-Measure = 0.91 (threefold CV)
Domestic Violence Crisis Identification From Facebook Posts Based on Deep Learning	Subramani et al.	2018	Facebook posts about "domestic violence and domestic abuse"	Binary Classification (Crisis—non-crisis)	LSTM-type models	Classification Accuracy = 0.94 (tenfold CV)
Child Abuse and Domestic Abuse: Content and Feature Analysis from Social Media Disclosures	Subramani et al.	2018	Facebook posts about domestic abuse and child abuse	Binary Classification (Abuserelated, General)	Decision Tree with Pscyholinguistic Features (LIWC)	Accuracy = 0.95; F1 = 0.94 (tenfold CV)
Intent Classification Using Feature Sets for Domestic Violence Discourse on Social Media	Subramani et al.	2017	Facebook posts from four Domestic Violence related Facebook groups	Binary Classification (Abuse – advice or support)	SVM with 15 selected LIWC features	Classification Accuracy = 0.97 (tenfold CV)
Quantitative Methods for Analyzing Intimate Partner Violence in Microblogs: Observational Study	Homan et al.	2020	#WhyIStayed and #WhyILeft Tweets	Binary Classification (#WhyIStayed—#WhyILeft)	Radial Basis Function SVM	Accuracy = 0.78
The Hidden Pandemic of Family Violence during COVID-19: Unsupervised Learning of Tweets	Xue et al.	2020	COVID-19 and Domestic Violence related Tweets in English	Unsupervised Learning (Cluster Analysis)	Latent Dirichlet Allocation (LDA)	N/A—unsupervised classification
Automated Identification of Domestic Violence in Written Child Welfare Records: Leveraging Text Mining and Machine Learning to Enhance Social Work Research and Evaluation	Victor et al.	2021	Records of referrals of child maltreatment from Michigan, USA	Binary Classification (DV—non-DV)	K Nearest Neighbours (KNN) Model, k = 30	Classification Accuracy = 0.91 (fivefold CV)
Knowledge Discovery in Databases—Amsterdam-Amstelland Police Force Research Project	Poelmans et al. Collected Studies	2008–2013	Dutch police reports of violent events from the AmsterdamAmstelland Police Force	Knowledge Discovery	Rule-based model developed using Formal Concept Analysis in combination with a Emergent Self Organising Maps visualisation; SVM and KNN with ESOM-enhanced inputs	Accuracy = 0.91 for incoming police reports; Accuracy = 0.89 for existing police reports; Accuracy = 0.95 for existing reports
Text-Mining Police Reports from New South Wales Project	Karystianis et al. Collected Studies	2019–2022	Police-attended domestic violence event narratives from New South Wales, Australia	Information Retrieval. Identify mentions of: mental health conditions, abuse types and injury type. Generate descriptive statistics on demographics	Rule-based dictionary model, IBM SPSS Statistics	Precision/F1 = 0.9/0.89 for types of abuse, 0.85/0.86 for victim injuries

The *N* = 22 included studies cover a wide range of research questions and text mining methodologies. Outcomes include extracting topics from a corpus of social media texts (More & Francis, 2021; Rodriguez & Storer, 2020; Xue, Chen, Chen, Hu, & Zhu, 2020; Xue et al., 2019), information retrieval of abuse and injury types from police reports (Adily et al., 2021), detecting the presence or absence of mentions of domestic violence in various types of text (Allen, Davis, & Krishnamurti, 2021; Botelle et al., 2022; Victor et al., 2021), and event and entity recognition from court documents (Li, Sheng, Ge, & Luo, 2019) and victim-survivor narratives (Liu, Li, Liu, Zhang, & Si, 2019). A summary of the studies can be found in Table 1.

The quantity of this research seems to be increasing in recent years, with the majority (*n* = 18) of studies being published in the last 5 years, and almost a third (*n* = 7) being published in the last two years. This may be a reflection the increased public awareness of the ‘shadow pandemic’ of domestic abuse brought on by the COVID-19 pandemic (Xue et al., 2020). Given the interdisciplinarity of the topic, it is interesting to note that there was an equal split between studies published in computer science journals and conferences^2^ (*n* = 11), and those published in social science and health related venues^3^ (*n* = 11).

The following section reviews the included studies as follows: firstly, by giving an overview of the different text mining models and techniques used in the studies; secondly, by reviewing the characteristics of the various datasets which studies used; and finally, by discussing how studies evaluated their techniques and models and what the evaluation outcomes were. This is followed by the Discussion section which investigates the quality of the included studies, offers lessons for researchers hoping to use text mining in their own work, considers ethical concerns of using computational text mining in the study of IPV, and examines the limitations of the current review.

### Models and Techniques

#### Supervised Techniques

Supervised techniques are those that are developed using a *labelled* dataset – a dataset where each instance has been annotated (labelled) with an outcome or category (for example, each Tweet in a Twitter corpus is manually labelled with either ‘about domestic abuse’ or ‘not about abuse’). These existing annotations can be used as a benchmark to evaluate automatic text mining methods, which makes supervised techniques a popular choice. The majority (*n* = 16) of the included studies used some kind of supervised approach. Supervised techniques are also the basis for many ML models. Supervised ML models ‘learn’ patterns from the labelled dataset to create an accurate model that can then be applied to new, unseen data (Alpaydin, 2020). This is an extremely convenient way to extend classification tasks to a dataset that is much larger than could be annotated by hand (Botelle et al., 2022).

There are two broad types of Supervised ML models: Traditional and Deep Learning models. Traditional models, such as Support Vector Machines (SVMs), K-Nearest Neighbours (KNN), LASSO Regression, and Decision Trees (DTs) iteratively try to find the best fit for the boundaries between one or more *classes*^4^ in a high dimensional space—a process commonly referred to as model *training*. It is beyond the scope of this paper to explain the mechanisms behind these algorithms, but clear introductory explanations can be found in Prabakaran et al. (Prabakaran, Waylan, & Penfold, 2017). In over a third of the included studies (*n* = 8) a Traditional Supervised ML model was the main, or most successful, approach (Allen et al., 2021; Chu et al., 2021; Garrett & Hassan, 2019; Homan et al., 2020; Schrading, Alm, Ptucha, & Homan, 2015; S. Subramani, Vu, & Wang, 2017; S Subramani, Wang, Islam, Ulhaq, & O’Connor, 2018; Victor et al., 2021), with SVMs being the most common successful model (Garrett & Hassan, 2019; Homan et al., 2020; Schrading et al., 2015; S. Subramani et al., 2017).

Deep Learning models were used as the main approach in six studies (Botelle et al., 2022; Karystianis et al., 2021; Li et al., 2019; Liu et al., 2019; S. Subramani et al., 2019; S. Subramani et al., 2018a, 2018b), often using a traditional ML model as a comparator baseline. Deep Learning models are very large networks of decision nodes – known as neural networks – which discover extremely complex multi-dimensional relationships between input and output (Alpaydin, 2020). Convolutional Neural Networks (CNNs) and Recurrent Neural Networks (RNNs) are two broad families of Deep Learning models (S. Subramani et al., 2019). Long Short-Term Memory (LSTM) models are an extension of RNNs often used for text classification tasks (S. Subramani et al., 2019).

Transformer based models, such as BERT (Devlin, Chang, Lee, & Toutanova, 2018), are very large deep models that have already learnt a statistical representation of a language (most commonly, English) from huge amount of data. For instance, the original BERT model was trained on a corpus of books and Wikipedia entries of over 3 billion words (Devlin et al., 2018). Since these *pre-trained* models have a wide ‘understanding’ of language already, they are very adaptable to new tasks, even those where there is little data available. One included study used BioBERT (Lee et al., 2020), an adaptation of the original BERT model specifically suited for biomedical text mining tasks, to identify instances of IPV in Electronic Health Records (Botelle et al., 2022).

Deep Learning models often achieve better results than Traditional ML in complex tasks (Botelle et al., 2022; S. Subramani et al., 2018a, 2018b). However, their drawback is their high level of opacity, which explains why they are frequently referred to as ‘black boxes’. Processes like *feature ablation*^5^ (Karystianis et al., 2021) and *dimensionality reduction*^6^ (S. Subramani et al., 2019) can help to visualise and understand the most important factors in the decision of a model. Additionally, recent advances in the domain of *explainable machine learning* have resulted in tools such as Local Interpretable Model-Agnostic Explanations (LIME) (Ribeiro, Singh, & Guestrin, 2016) which can be used to provide insight into the decision-making mechanisms of Deep Learning models. Nonetheless, their results can still prove difficult to interpret (Karystianis et al., 2021).

The remaining two studies which used a supervised approach used rule-based models to automatically classify data, using existing labels to test the accuracy of their rules (Karystianis et al., 2022; J Poelmans, Van Hulle, et al., 2011). Hand-crafted rule-based models have the advantage of being very transparent and efficient in comparison to ML models. It is probably not a coincidence that the two studies which used this approach were both actively working with police forces, who are likely to value transparency highly. Rule-based models performed very well in both studies (0.89 F1-score for abuse types (Karystianis et al., 2022); Accuracy > 0.89 for identifying domestic violence in police reports (J Poelmans, Van Hulle, et al., 2011)). This suggests that they should not be overlooked in favour of more modern but complex tools such as Deep Learning models.

#### Unsupervised Techniques

Six studies used unsupervised topic modelling or exploration as their primary approach (More & Francis, 2021; Rodriguez & Storer, 2020; Sanchez-Moya, 2017; Xu, Zeng, Tai, & Hao, 2022; Xue et al., 2020; Xue et al., 2019). Here we use ‘unsupervised’ to mean that a dataset has no labels or annotations—it is simply a collection of instances of raw text data (for example, a collection of Tweets *without* any categories or labels assigned to each Tweet).

##### Unsupervised Clustering

Four of the six studies used Unsupervised Machine Learning (Unsupervised ML) models, which analyse the latent structure of a text corpus to identify related clusters, or *topics*, in a process called *topic modelling*. The most common topic modelling approach was Latent Dirichlet Allocation (LDA), used in three studies (More & Francis, 2021; Xue et al., 2019, 2020), whilst the other study used Structural Topic Modelling (STM) (Rodriguez & Storer, 2020).

##### Unsupervised Exploratory Approaches

Two studies used forms of exploratory data analysis as their primary method of investigating text data. Xu et al. (Xu et al., 2022) deployed a custom rule-based approach to *sentiment analysis.* The latter describes the practice of analysing texts according to their positive or negative emotional tone.

Sanchez-Moya (2017) used Linguistic Inquiry and Word Count (LIWC) (Pennebaker, Francis, & Booth, 2001), a computational tool for linguistic analysis. This technique was also used in four other studies as an addition, or an input into, more complex models (Allen et al., 2021; Rodriguez & Storer, 2020; S Subramani et al., 2018a, 2018b). LIWC is a *dictionary*-*based* method, in that it counts the number of words in a text which belong to a series of dictionaries of words from particular linguistic categories (e.g. positive affect, negative affect, biological processes, analytical thinking, emotional tone) (Sanchez-Moya, 2017). Dictionary-based methods are a simple but powerful instrument than can be very efficient, and used across multiple studies, once the hurdle of creating the initial dictionary has been passed. Other included studies created their own dictionaries of IPV-related terms (Adily et al., 2021; Li et al., 2019; J. Poelmans, Elzinga, Viaene, & Dedene, 2009).

#### Technologies

Matlab, R and Python were mentioned most often as technologies used in the studies, reflecting their popularity for data science applications. At least seven studies mentioned using Python (Chu et al., 2021; Garrett & Hassan, 2019; Homan et al., 2020; More & Francis, 2021; Schrading et al., 2015; Xu et al., 2022; Xue et al., 2019), although many studies did not report any specific technology or programming language used.

### Datasets

#### Source

Most of the datasets used in the included studies were sourced from social media (*n* = 15) with the remainder coming from police forces (*n* = 3), health services (*n* = 1), litigation proceedings (*n* = 1), children’s social workers (*n* = 1), and a single study which directly recruited participants (*n* = 1). A summary of the datasets can be found in Table 2.

**Table 2:** Datasets Used in Included Studies

Dataset	Authors	Year	Type	Source	Perspective	Geography	Language	Size	Labels
Chinese social media comments about Yuya IPV disclosure	Xu et al.	2022	Social Media posts	Chinese Social Media	Mix	Chinese-speaking world	Chinese	34,350	N/A
Electronic Health Records (EHRs) from the South London and Maudsley NHS Foundation Trust	Botelle et al.	2022	Electronic Health Records	South London and Maudsley NHS Foundation Trust	3rd Party	UK	English	5,282	Manual—2 Student Reviewers, weekly labelling meetings
English-Language Reddit posts, Tweets and news articles discussing domestic abuse	More and Francis	2021	Social Media posts	Reddit, Twitter and News	Mix	English-speaking world	English	Unspecified	N/A
Police-attended domestic violence event narratives from New South Wales, Australia	Karystianis et al. Collected Studies	2019–2022	Police Reports / Mental Health Data	New South Wales Police Force, Australia	3rd Party	Australia	English	492,393	Existing—internal police officer classification process
English-Language Tweets tagged with #WhyIstayed or #WhyILeft	Rodriguez and Storer	2020	Social Media posts	Twitter	Mix	English-speaking world	English	3060	Automatic—According to Hashtags
Tweets about Domestic Violence	Xue et al.	2019	Social Media posts	Twitter	Mix	English-speaking world	English	322,863	N/A
Safecity English-language narratives of sexual harassment incidents (originally published by Karlekar and Bansal)	Liu et al.	2019	Social Media posts	Safecity social media platform	1st Person Reported	English-speaking world	English	9,892	N/A
Chinese-Language litigation texts from divorce proceedings	Li et al.	2019	Litigation Texts	Divorce proceedings	3rd Party	China	Chinese	3,100	Manual—Events annotated according to Beginning-Inside Outside (BIO) method
Tweets using the #WhyIDidntReport hashtag from US cities	Garrett and Hassan	2019	Social Media posts	Twitter	Mix	USA	English	37,526	N/A
British domestic violence charity online forum posts	Sanchez-Moya	2017	Social Media posts	British Domestic Violence Charity Forum	Mix	UK	English	472	Automatic—According to forum topic
Reddit posts discussing abuse and not relevant to abuse	Schrading et al.	2015	Social Media posts	Reddit	Mix	English-speaking world	English	370,410	Automatic—According to forum topic
Diary entries of pregnant women	Allen et al.	2021	Diary Entries	Diary Entries from pregnant women	1st Person Reported	USA	English	309	Automatic—according to psychometric measures
Chinese-Language posts from Baidu Teiba group about intimate partner violence	Chu et al.	2021	Social Media posts	Baidu Teiba	Mix	Chinese-speaking world	Chinese	4,800	Manual—2 Student Reviewers
Facebook posts relating to domestic violence	Subramani et al.	2019	Social Media posts	Facebook	Mix	English-speaking world	English	1,654	Manual—2 Student Reviewers
Facebook posts about "domestic violence and domestic abuse"	Subramani et al.	2018	Social Media posts	Facebook	Mix	English-speaking world	English	2,060	Manual—Multiple reviewers
Facebook posts about domestic abuse and child abuse	Subramani et al.	2018	Social Media posts	Facebook	Mix	English-speaking world	English	4,239	Automatic—According to search term
Facebook posts from four domestic violence related Facebook groups	Subramani et al.	2017	Social Media posts	Facebook	Mix	English-speaking world	English	8,856	Manual—2 Student Reviewers
#WhyIStayed and #WhyILeft Tweets	Homan et al.	2020	Social Media posts	Twitter	Mix	English-speaking world	English	17,534	Manual—4 student reviewers
COVID-19 and domestic violence related Tweets in English	Xue et al.	2020	Social Media posts	Twitter	Mix	English-speaking world	English	1,015,874	N/A
Records of referrals of child maltreatment from Michigan, USA	Victor et al.	2021	Case Summaries	Records of child maltreatment in Michigan, USA	3rd Party	USA	English	75,809	Manual—4 student reviewers
Dutch police reports of violent events from the Amsterdam-Amstelland Police Force	Poelmans et al. Collected Studies	2008 -2013	Police Reports	Amsterdam-Amstelland Police Force	3rd Party	The Netherlands	Dutch	9,552	Existing—internal police officer classification process

As expected from a search conducted in English, most datasets (*n* = 18) are in English, with the others being in Chinese (*n* = 3) and Dutch (*n* = 1). Of those datasets sourced from a particular locality (e.g. police data), the US, UK, Australia, China and the Netherlands are represented. Datasets are notably missing from other countries where English is widely spoken, such as Canada, India, Pakistan, South Africa or Nigeria. Around a quarter of the datasets (*n* = 6) describe abuse from the perspective of a 3^rd^ party reporting on the abuse (e.g. a police officer or healthcare professional). Conversely, a small number (*n* = 2) describe abuse from the perspective of the victim-survivor narrating their own experience(s). The remaining datasets (*n* = 14) contain a mix of perspectives (e.g. social media groups where some posts are from the victim-survivor perspective and some are from 3^rd^ parties describing abuse which happened to someone else or offering support). No datasets explore either text written from the perspective of a perpetrator, or direct evidence of abuse in text (e.g. abusive text messages).

#### Size

The size of the datasets varies considerably, from 309 diary entries (Allen et al., 2021) to over 1 million unique Tweets (Xue et al., 2020). The size of each text within a dataset also varies, from a single Tweet (Homan et al., 2020) to entire litigation texts (Li et al., 2019) or case summaries (Victor et al., 2021). Of the datasets used for supervised ML tasks, the average size was 73,847 instances.

#### Labelling Process

Data labelling is often a time consuming and costly part of computational text mining, which can discourage research from taking place in new areas. In addition, data labelling has a direct impact on the outcome of classification models, since any bias or inaccuracies in the labelling process are likely to be picked up and replicated by the model (Bechmann & Zevenbergen, 2019; Dignum, 2017). For this reason, accurate and transparent labelling is of paramount importance, especially in sensitive research.

Most datasets were labelled by supervised student reviewers. However, some datasets took advantage of existing properties of the data to create labels – for example, by using hashtags applied to tweets (Homan et al., 2020), participant surveys administered alongside the collection of text data (Allen et al., 2021), or police assigned labels collected during the incident reporting process (J Poelmans, Van Hulle, et al., 2011). Such techniques can significantly reduce the time and cost burden for researchers and show the benefit of trying to find label-type properties within existing data.

### Evaluation

#### Test and Train Set

A *test set* is a portion of the dataset that is set aside during model development, and subsequently used to evaluate the algorithm’s final performance on held-out data. Leaving part of the data out during model development helps avoid *overfitting*, where models learn the statistical characteristics of a dataset “too well”, in a way that means their results don’t generalise to other data (Arango, Pérez, & Poblete, 2019). For small datasets, a mechanism called *k-fold Cross Validation* (k-fold CV) is often used to evaluate a model’s performance, in combination with or instead of a separate test set. This involves separating the data into *k* different segments. The model is then allowed to see all but one of these segments when it is training, and after training has finished, the left-out segment is used to test the model. The process is then repeated k times, each time leaving out a different segment. The results of these k times are then averaged to give an overall evaluation metric.

#### Evaluation Metrics

All studies using supervised techniques were evaluated using a test set or k-fold CV. *Accuracy* and *F1 score* were the most common metrics used to report how well the model performed at correctly categorising the texts. Accuracy refers to the overall percentage of instances which were correctly classified. The F1 score is an alternative metric which balances *Precision* (also known as specificity, or true negative rate) and *Recall* (also known as sensitivity, or true positive rate). The F1 score is useful in situations where one class is much larger than another – in this case, Accuracy scores can be unhelpfully biased towards the dominant class (Rosa et al., 2019).

However, comparison of models across different datasets using reported metrics should be done cautiously, since much of the performance of a model depends on the data it was trained on. Some datasets simply have too much overlap between the characteristics of different classes, making it difficult for a model to distinguish between them.

Taking into account these comments on the limitations of metrics, there is a very wide range of accuracies in the studies, from 0.69 (which would usually be considered too low to be used in any practical application) (Karystianis et al., 2021) to 0.97 (as good of a performance as can reasonably be expected from most models) (Botelle et al., 2022). There was no single type of model or technique which performed well across the studies. This reflects the variability of model tasks within the studies and demonstrates the importance of choosing the right model for the task in question.

#### Unsupervised Evaluation

Evaluation of the studies which used unsupervised approaches was much more variable, reflecting the difficulties in evaluating unsupervised methods more broadly (Zhao et al., 2015). Some unsupervised studies did not include any explicit evaluation of their technique (Xu et al., 2022) or were using tools developed and tested in previous research (such as LIWC (Sanchez-Moya, 2017)). Other studies which used unsupervised topic modelling attempted to evaluate the optimal number of topics, using methods such as Rate of Perplexity Change (RPC) (Xue et al., 2019).

## Discussion

Overall, the *N* = 22 studies showcase different models and techniques which can be used for IPV research, as well as a variety of datasets and evaluation mechanisms. The quality of studies varied considerably across the included works—full results from the Quality Assessment (i.e. 21 ‘yes/no’ criteria) are reported in Table 3. This variation in quality reflects the innovative nature of this new, interdisciplinary area. There are not yet clear guidelines about how to use text mining methodologies in social science research. In addition, challenges arise when attempting to assess quality across such a heterogeneous set of studies. For example, some papers did not report any pre-processing steps (Criteria 6) since this is not useful in Deep Learning architectures (S. Subramani et al., 2018a, 2018b). Other studies did not report demographic characteristics of their dataset (Criteria 3) due to ethical concerns about collecting personal identifiers (Rodriguez & Storer, 2020; Xue et al., 2019).

**Table 3:** Quality of Studies

Author	1	2	3	4	5	6	7	8	9	10	11	12	13	14	15	16	17	18	19	20	21
Victor et al., 2021	✓	✓	✓	✓			✓			✓	✓		✓	✓		✓	✓	✓	✓	✓	
Botelle et al., 2022		✓					✓			✓							✓	✓	✓	✓	✓
More & Francis, 2021				✓	✓	✓	✓	✓	✓	✓	✓	✓					N/A	N/A	N/A	N/A	N/A
Schrading et al., 2015	✓	✓		✓		✓	✓			✓	✓	✓					✓	✓	✓	✓	✓
Karystianis et al. Col. Studies	✓	✓	✓	✓	✓	✓	✓	✓		✓	✓		✓			✓		✓	✓	✓	✓
Xu et al., 2022	✓	✓		✓	✓	✓	✓	✓	✓	✓	✓	✓	✓		✓	✓	N/A	N/A	N/A	N/A	N/A
Karystianis et al., 2021		✓		✓	✓	✓	✓		✓	✓	✓	✓	✓	✓		✓	✓	✓	✓	✓	
Rodriguez & Storer, 2020		✓		✓	✓	✓	✓			✓	✓	✓	✓	✓		✓	N/A	N/A	N/A	N/A	N/A
Xue et al., 2019				✓	✓	✓	✓	✓	✓	✓	✓	✓				✓	N/A	N/A	N/A	N/A	N/A
Liu et al., 2019		✓				✓	✓	✓		✓	✓	✓						✓	✓	✓	
Li et al., 2019		✓	✓	✓	✓	✓	✓	✓	✓	✓	✓	✓				✓	✓				
Garrett & Hassan, 2019	✓	✓		✓			✓										✓	✓		✓	✓
Sanchez-Moya, 2017	✓	✓	✓	✓	✓	✓	✓	✓	✓	✓	✓	✓	✓	✓		✓	N/A	N/A	N/A	N/A	N/A
Allen et al., 2021	✓	✓		✓		✓	✓	✓	✓	✓	✓		✓	✓		✓	✓	✓		✓	
Subramani et al., 2017					✓	✓	✓	✓	✓	✓	✓		✓				✓	✓	✓		
Subramani, Wang, Islam et al. 2018	✓	✓			✓	✓	✓	✓	✓	✓	✓	✓					✓	✓			
Subramani, Wang, Vu & Li., 2018a, 2018b	✓	✓			✓	✓	✓	✓	✓	✓	✓	✓	✓			✓	✓	✓	✓		
Subramani et al., 2019	✓	✓	✓	✓	✓	✓	✓		✓	✓	✓		✓	✓			✓	✓	✓		
Chu et al., 2021	✓	✓		✓	✓	✓	✓	✓	✓	✓	✓					✓	✓	✓	✓	✓	
Homan et al., 2020		✓		✓	✓	✓	✓						✓			✓	✓	✓	✓	✓	
Xue et al., 2020	✓	✓	✓	✓	✓	✓	✓	✓	✓	✓	✓	✓	✓			✓	N/A	N/A	N/A	N/A	N/A
Poelmans et al. Col. Studies	✓	✓	✓	✓			✓	✓		✓	✓		✓				✓	✓		✓	✓

The following section provides a more detailed discussion of the reviewed studies, focusing on lessons learned for future research, and issues of ethics and bias raised by using computational methods to research IPV.

### Lessons for Future Research

Examining aspects of the included studies offers lessons for future research, particularly regarding the definition of violence, open source code, and overall study design. These issues are discussed in more depth below.

#### Definition of Violence

The definition of violence is mentioned in just over half the studies (*n* = 13), but many do not define IPV at all, or very briefly reference a definition from another entity, such as the WHO (Chu et al., 2021). Studies tend to discuss the definition of violence in most detail when examining the dataset labelling process for supervised techniques. Labelling data often highlights conflicting definitions between annotators and necessitates a more in-depth description of what constitutes violence (Botelle et al., 2022; J. Poelmans, Elzinga, Viaene, & Dedene, 2009). Considering wider difficulties defining IPV within research (Alhabib et al., 2010; Barocas et al., 2016), future researchers should ensure they carefully describe and motivate the specific definition of IPV used in their work.

#### Open Source

Unfortunately, no projects in the study reported that their code was *open source*. The latter describes a trend in computer science to make code and data available freely online, to facilitate collaborators wishing to build similar applications.^7^ Only two projects mentioned that their dataset would be made available upon request (Botelle et al., 2022; Xu et al., 2022). This is perhaps unsurprising when it comes to datasets, given the sensitive nature of the data involved. However, future work could consider making source code available for other researchers, to encourage knowledge-sharing within this field.

#### Study Design

In general, future projects could consider a number of factors in study design. Firstly, researchers may reflect where novel data can be sourced, and whether data from multiple sources can be joined-up for additional insight (Karystianis et al., 2021). Secondly, once a model has been developed, researchers could consider deploying or testing it in an active service-provision environment. For example, research projects from Poelmans et al. (2013) and Karystianis et al. (2022) successfully worked with police forces to implement knowledge-discovery techniques within their day-to-day operations, and models revealed edge cases of abuse that the police had previously missed (Hwang et al., 2020; J. Poelmans, Elzinga, Viaene, & Dedene, 2009). A project to detect sexual and physical domestic violence in Electronic Health Records is now live on systems of an NHS trust in the UK (Botelle et al., 2022).

Moreover, when designing methodologies, researchers must consider more than just the choice of model. Rule-based, Traditional ML, Deep Learning and Unsupervised approaches all performed well in different included studies, demonstrating that the context and appropriateness of a model is more important than its type. The importance of initial data exploration and feature selection should not be ignored, as these processes (referred to as *feature engineering*^8^) significantly increase the quality of outcomes. For example, Subramani et al. (2017) did not use the raw text, but instead the outcome of LIWC (see Unsupervised Exploratory Approaches, above), as the input to their ML model (S. Subramani et al., 2017). Finally, several studies highlighted the importance of mixed methods in their research, and the significance of pairing quantitative methods with qualitative insights (Rodriguez & Storer, 2020; Victor et al., 2021).

### Ethical Concerns and Bias

#### Ethics and Context

In general, little attention was paid to ethics across the studies, with only six publications including an explicit ethical discussion. However, a large number (*n* = 14) of studies do mention limitations of their work or discuss appropriate contexts for model use. For example, Victor et al. indicate that whilst their model performs well enough to be used for generating accurate descriptive statistics about domestic violence in a dataset of child welfare case summaries, it would be inappropriate for use in decision making about individual cases (Victor et al., 2021). They highlight the importance of qualitative analysis when using ML methods in an interdisciplinary context, giving three examples of how qualitative analysis can enrich ML research in this domain: understanding the data-generating mechanism, its context, content and what inferences can reasonably be made; understanding outliers and misclassifications in order to improve the model; and applying insights from the model to help standardize the assessment or documentation of abuse (Victor et al., 2021).

#### Bias

Allen et al. comment on the lack of diversity in their sample, which contained mostly white participants (Allen et al., 2021). Since non-white groups may be more likely to experience IPV (Breiding, Chen & Black, 2014), this lack of diversity is especially troubling. However, very few studies commented on the demographic representativeness of their dataset and whether downstream applications built on their models risked bias towards certain groups.

#### Future Work

Given the recent emphasis within ML communities on ethical principles of accountability, responsibility and transparency (Dignum, 2017; Floridi et al., 2018), future work must take more of a focus on discussing the foundational ethical questions raised by this kind of research. Researchers might consider following ethical guidelines for ML such as those proposed by the Association of Internet Researchers (Bechmann & Zevenbergen, 2019). The consequences of ignoring such ethical discussions are significant: At their worst, ML models could contribute to the invalidation and minimisation of different experiences of abuse, for example by classifying an instance as ‘not abuse’ and leading to a victim-survivor not receiving services or justice after having experienced great harm (Blackwell, Dimond, Schoenebeck, & Lampe, 2017). Victim-survivors of IPV have experienced situations in which they have had their opinions and experiences repeatedly invalidated, belittled, denied and manipulated (Stark, 2009). Researchers must be aware of the potential mis-use of their research to extend this denial of the victim-survivor’s reality. Models are representations of reality, but they are not reality themselves, and the way text mining research is conducted and presented should reflect this understanding.

### Limitations

The current work is subject to several limitations. Firstly, since the search strategy only included academic literature, it is possible that important grey literature may have been missed. Secondly, the search terms included other types of violence such as “family violence” and “sexual violence”, aiming to capture all definitions of violence that may include IPV. Some of the reviewed studies may therefore have included incidents of non-partner abuse in their data. Finally, the Quality Assessment criteria used in the review were developed by combining multiple existing methods and were not thoroughly evaluated on different types of studies outside this review. They should therefore not be used as a ranking mechanism or to draw concrete conclusions about the quality of individual studies.

## Conclusion

Twenty-two studies which used computational text mining to investigate IPV were identified through a systematic literature review of eight academic databases. The studies included datasets from social media, police forces, a healthcare provider, and social work and legal settings. A variety of supervised and unsupervised text mining techniques were used on these datasets for tasks which included detecting the presence or absence of IPV as well as identifying abuse types, extracting entities and events, or understanding themes. Some studies commented on the ethics or real-world deployment of their findings, but future research could include more in-depth discussion of these. Additionally, potential areas for future work may include sourcing datasets from other geographies and types of organisations, explorations into sub-types of abuse, plus the application of emerging text-mining methods in the IPV field as they develop.

## Footnotes

1 NB The search string was adapted to fit the search functions of different databases. In ArXiv.org, only the IPV-related part of the search string was used since all research on ArXiv.org was assumed to have a computational element and the search function did not allow for so many search terms.

2 e.g. NAACL, IEEE Transactions, Databases Theory and Applications.

3 e.g. Violence Against Women, Journal of Interpersonal Violence, Journal of Medical Internet Research.

4 In ML literature, *class* refers to an outcome, category, or label that a model is trying to optimise for. For example, if a model was being built to automatically categorise (or *classify)* Electronic Health Records as to whether or not they contained a mention of domestic abuse, the two *classes* would be “abuse present” and “abuse absent”.

5 Feature ablation refers to removing one or more inputs (*features*) and observing the change in model performance in order to understand how different features affect the decision of a model.

6 Dimensionality Reduction refers to the mathematical process of transforming a very high-dimensional space into a space with fewer dimensions, whilst preserving important characteristics of the data. This can help to visualise clusters or decision boundaries within a complex model.

7 This can be done using free services such as GitHub (https://github.com), GitLab (https://about.gitlab.com), Zenodo (https://zenodo.org) or Dataverse (https://dataverse.org).

8 Feature engineering is the set of steps that transform raw data into numeric values that are usable by ML models. These numbers are features that represent each instance/sample (e.g. an abusive sentence or a non-abusive one) and are used as inputs by the ML models.
